# Toward a bioethical issue: induced multiple pregnancies and neonatal outcomes

**DOI:** 10.1186/1824-7288-36-74

**Published:** 2010-11-11

**Authors:** Antonio A Zuppa, Giovanni Alighieri, Piero Catenazzi, Antonio Scorrano, Costantino Romagnoli

**Affiliations:** 1 Department of Pediatrics, Division of Neonatology, Catholic University of the Sacred Heart, Largo Gemelli, 00168 Roma, Italy

## Abstract

Assisted reproductive technology has made great progress during the last three decades. After the initial enthusiasm, many ethical, legal and social issues related to the application of these procedures began to evolve. Multifetal pregnancy and fetal reduction, embryo cryopreservation, preimplantation genetic diagnosis, risks of birth defects and other adverse outcome associated with assisted reproductive technology are issues that have to be addressed building future collaborative studies and continuing the debate on related ethical issues.

## Summary

The rapid evolution of ART has revealed certain ethical issues that have to be addressed such as multifetal pregnancy and fetal reduction, embryo cryopreservation, preimplantation genetic diagnosis, risks of birth defects and other adverse outcome associated with assisted reproductive technology.

Advances in human reproductive biology during the last three decades enabled the increased use of ovulation induction and the introduction and rapid progress of assisted reproductive techniques (ART), defined as any procedure that entails the handling of both eggs and sperm or of embryos for the purpose of establishing a pregnancy (i.e., in vitro fertilization -IVF-, intracytoplasmatic sperm injection -ICSI-) [1].

As consequence, the incidence of multiple pregnancies has increased: twin births have doubled and the number of triplet births has tripled [2]. In the 1990s, the ART and non-ART technologies were responsible for at least two thirds of all multiple pregnancies, and for the majority of high order multiple pregnancies [3].

Because of pressure from politicians and international societies it is hard, in Europe, to find countries with the same rules regarding medically assisted reproduction [4].

International studies in the last ten years have continued to show an increased incidence of preterm birth (<37 weeks' gestation), low birth-weight (<2500 g) and associated adverse neonatal outcomes in ART births compared with naturally conceived births [3,5-8].

In an interesting review it was found a difference in singleton pregnancies outcomes between natural and assisted conceptions with a worse perinatal outcome in the second one. It was not similarly observed in assisted twin pregnancies that seems to have outcomes that are either similar to or slightly better than those conceived naturally. But this is poor consolation for the much greater risks of twin pregnancy overall. Virtually all perinatal and infant morbidity occurs more frequently in twins than in singletons. One of the most results of this study is that there is an increased perinatal mortality in singleton and twin pregnancies after assisted conception than in natural conception [9].

In our cohort study we compared 228 neonates from spontaneous twin pregnancies with 32 neonates from induced twin pregnancies, showing a significantly higher incidence of prematurity, low birth-weight, severe depression at birth and respiratory disease in the latter [10].

More recently, we conducted another cohort study comparing 6 spontaneous triplet pregnancies with 18 induced triplet pregnancies [11]. In spite of the lack of significant differences between the two groups, the assisted reproduction group showed more complications. According to international data, the results suggest that the incidence of major neonatal morbidity (i.e., neonatal malformations) might increase due to assisted reproduction.

Additionally, there has been a suggestion that ART births have a small but significantly increased incidence of birth defects. Rates of ART-associated birth defects are 1.4 to 2.0 fold higher than the overall rate of 3% to 4% of births in general [12]. A large study from Western Australia examined 301 IVF infants, 837 ICSI infants, and 4,000 naturally conceived controls [13]. The authors found an unadjusted odds ratio of developing congenital birth defects of 2.2 (1.3 to 3.3) for ICSI and 2.6 (1.7 to 3.0) for IVF compared with controls. On adjustment for multiple gestations and maternal age and parity, the odds ratios remained significantly elevated at 2.0 (1.3 to 3.2) and 2.0 (1.5 to 2.9) for ICSI and IVF, respectively. Some authors have reported in infants conceived with ART small increases in specific birth defect rates, such as neural tube defects, omphaloceles and hypospadias [14].

ART births are also associated with an increased incidence of chromosomal abnormalities and imprinting defects, as Beckwith-Wiedemann Syndrome, Angelman Syndrome, Silver-Russel Syndrome, Maternal Hypomethylation Syndrome and Retinoblastoma [15]. Regarding to chromosomal abnormalities, a meta-analysis compared ICSI conceived fetal karyotypes with those in the normal neonatal population and documented an increased risk of de novo anomalies and inherited chromosomal defects, usually from an infertile father [16]. This risk estimates among women receiving ART is readily confounded by overlapping risk factors including multiple pregnancies, underlying causes of infertility and factors associated with ART themselves (i.e., the avoidance of natural selection mechanism of sperm during the course of a natural conception, the delayed fertilization of the oocyte, the freezing and thawing of embryos) [17,18].

As regards imprinting defects, ART procedures including ovarian stimulation and the manipulation of preimplantation embryos occur during critical developmental periods when genomic imprints have been shown to be vulnerable in animal studies. The defect more frequently observed involves DNA methylation, especially loss of maternal methylation that seems to be due to underlying subfertility or ovarian stimulation without subsequent in vitro procedures [15,19].

However, these findings require further confirmation because it would be very difficult to design randomised controlled trials to study the effects of ART and non-ART technologies with natural conception. Much of the information relies on observational studies or small cohort studies that may not have significant power.

Developments of the ART over the last thirty years have created unexpected public interest in certain aspects of human reproduction. After the initial enthusiasm, many ethical, legal and social issues related to the application of these procedures began to evolve, which led to serious discussions and often disagreements among the involved physicians, public and the state itself: multifetal pregnancy and fetal reduction, embryo criopreservation, preimplantation genetic diagnosis, genetic material donation and surrogacy [20].

Legislations and guidelines for infertility clinics have been outlined, along with strategies to limit the number of embryos transferred to achieve a lower risk of multiple births. Since 1997, a decrease has occurred in the number of embryos transferred and the percentage of gestation with three or more fetuses [21].

The common practice of physicians is to transfer to the uterus only two or three embryos in any cycle, although many embryos are produced during a single IVF cycle. Then human embryo cryopreservation has become integral part of ART and there is little knowledge about the limits of storage period and the possible effects of long term storage.

Until advances in assisted reproductive technology eliminate the iatrogenic cause of multiple gestation, fetal reduction offers hope for a good outcome in an otherwise adverse situation, such as a multiple pregnancy where its continuation represents a threat to the life or health of the mother.

The recent advances in genetic disorders have made possible to diagnose the genetic conditions in the embryos before implantation in a setting of in vitro fertilization. Polymerase chain reaction and fluorescence in situ hybridization are the two common techniques employed on a single or two cells obtained via embryo biopsy [22].

It is our view that several approaches are needed to better address real risk for ART complications: guidelines on the number of embryos that should be transferred, detailed information on the use of specific ART techniques on birth certificates, ART registry data, the linkage of the latter to birth defects registry data, prospective studies of ART births.

Obstetricians and pediatricians need to become sources of such information. Couples who want to use ART should be counselled about the risk/benefit associated with these techniques. An educated counsel is needed because evidence reveals that the diagnosis of infertility itself may increase the risk of perinatal complications.

In spite of the developments in reproductive medicine and the changes that have taken place to the structure of the society, a number of medical and ethical issues still remain unresolved.

Furthermore socioeconomic concerns are also important if we consider the remarkable use of human and technological resources needed to guarantee a good outcome in an induced multiple pregnancy.

In a pluralistic society it is more problematic to reach consensus on universal policy about assisted reproduction.

Would be acceptable to set the limits for the provision of these very useful treatments?

This open question must be addressed building future collaborative studies and continuing the debate on related ethical, legal and social issues.

## Competing interests

The authors declare that they have no competing interests.

## Authors' contributions

AAZ conceived of the study, and participated in its design and coordination. GA wrote the manuscript and participated in the design of the study. PC wrote the manuscript. AS wrote the manuscript and participated in the design of the study. CR designed the study. All authors read and approved the final manuscript.
